# Conjoint research of WGCNA, single-cell transcriptome and structural biology reveals the potential targets of IDD development and treatment and JAK3 involvement

**DOI:** 10.18632/aging.205289

**Published:** 2023-12-12

**Authors:** Yingjing Zhao, Yuxue Mu, Yujia Zou, Zhijian He, Tianxing Lu, Xinhui Wang, Weihang Li, Bo Gao

**Affiliations:** 1 Department of Critical Care Medicine, Nanjing First Hospital, Nanjing Medical University, Nanjing 210006, Jiangsu Province, China; 2 Department of Orthopedic Surgery, Xijing Hospital, Air Force Medical University, Xi’an, China; 3 Aerospace Clinical Medical Center, School of Aerospace Medicine, Air Force Medical University, Xi’an, China; 4 Department of Cardiology, Xinhua Hospital affiliated to School of Medicine, Shanghai Jiaotong University, China; 5 Department of Sports Teaching and Research, Lanzhou University, Lanzhou, China; 6 Zonglian College, Xi’an Jiaotong University, Xi’an, Shaanxi, China; 7 Department of Oncology, The Fifth Affiliated Hospital of Xinxiang Medical College, Xin Xiang 453100, China

**Keywords:** intervertebral disc degeneration, weighted gene co-expression network analysis, Janus kinase family, high-throughput virtual screening, single-cell transcriptome

## Abstract

Objectives: This study conducted integrated analysis of bulk RNA sequencing, single-cell RNA sequencing and Weighted Gene Co-expression Network Analysis (WGCNA), to comprehensively decode the most essential genes of intervertebral disc degeneration (IDD); then mainly focused on the JAK3 macromolecule to identify natural compounds to provide more candidate drug options in alleviating IDD.

Methods: In the first part, we performed single-cell transcriptome analysis and WGCNA workflow to delineate the most pivotal genes of IDD. Then series of structural biology approaches and high-throughput virtual screening techniques were performed to discover potential compounds targeting JAK-STAT signaling pathway, such as Libdock, ADMET, precise molecular docking algorithm and *in-vivo* drug stability assessment.

Results: Totally 4 hub genes were determined in the development of IDD, namely VEGFA, MMP3, TNFSF11, and TIMP3, respectively. Then, 3 novel natural materials, ZINC000014952116, ZINC000003938642 and ZINC000072131515, were determined as potential compounds, with less toxicities and moderate ADME characteristics. *In-vivo* drug stability assessment suggested that these drugs could interact with JAK3, and their ligand-JAK3 complexes maintained the homeostasis *in-vivo*, which acted as regulatory role to JAK3 protein. Among them, ZINC000072131515, also known as Menaquinone, demonstrated significant protective roles to alleviate the progression of IDD *in vitro*, which proved the nutritional therapy in alleviating IDD.

Conclusions: This study reported the essential genes in the development of IDD, and also the roles of Menaquinone to ameliorate IDD through inhibiting JAK3 protein. This study also provided more options and resources on JAK3 targeted screening, which may further expand the drug resources in the pharmaceutical market.

## INTRODUCTION

Intervertebral disc degeneration (IDD) is mainly considered as the trigger of low back pain (LBP), affecting global medical burden and enormous socio-economic costs, which could cause severe life quality of patients [1, 2]. Currently, the clinical therapy regarding IDD mainly includes bed rest, medication treatment and surgical approach, while these methods could only relieve the symptoms but failed to reconstruct the homeostasis of intervertebral disc (IVD) [3, 4]. Served as family member of non-receptor protein-tyrosine kinases, Janus kinases (JAKs) consists of JAK1, JAK2, JAK3, and tyrosine kinase-2 (TYK2), they behave pivotal roles in cytokine pathways and are closely related to both inflammatory and neoplastic diseases [5]. The phosphorylate sites of JAKs could generate signaling effectors, mainly signal transducers and activators of transcription (STATs), which further cause the downstream signaling cascade and eventually lead to various diseases [6, 7].

Recently, the studies of JAKs family have attracted much attention, current research has reported the connections between JAK inflammatory signaling pathway and oxidative stress, that JAK/STAT signaling pathway is activated by ROS, and the process could be reversed by JAK inhibitors such as AG490 and ruxolitinib [8, 9]. Besides, the JAK/STAT signaling pathway in IDD have just received recent attention, existed studies have reported the inhibition of JAK/STAT signaling pathway could ameliorate the progression of IDD [10, 11]. JAK1, JAK2 and TYK2 in the JAKs family are reported to express ubiquitously in body, which mainly exist in various tissues, while JAK3 is almost highly expressed in hematopoietic tissues, which mainly present in bone marrow cells, thymic cells, NK cells and activated T and B lymphocytes [12]. Increasing evidence has proved the pivotal cell regulatory roles of JAK3, which chiefly mediate and participate in the formation of cytokines; thus the absence and disorder of JAK3 would lead to the dysfunction of lymphocytes and finally cause the loss of immuno-modulatory functions [13, 14]. The expression of JAK3 is also upregulated with a strong activity in the B-lineage acute lymphoblastic leukemia patients [15, 16]; besides, JAK3 protein site mutation is reported to be the regulatory gene in the development of different disorders [7, 17–20]. These above findings all imply that JAK3 is the key regulatory gene and signaling transduction member whether in the tumor or non-tumor disorders, which could be regarded as pharmacological target for these diseases to discover novel natural materials based on genetic therapy.

Currently, a highly selective covalently reversible JAK3 inhibitor is discovered in the JAKs family, namely FM-381 [21]. Natural products and their derivatives provide a great contribution to the high-throughput screening and design of potential lead drug skeletons, which are the most aspect of current pharmacological market, due to their unique chemical structures as well as biological functions [22–24]. Researches have reported that natural products were used for the treatment of various ailments since ancient times, and historically, huge number of new drug compounds were generated and extracted from nature [25, 26]. Untapped biological resources, ‘smart screening’ method, metabolic engineering, synthetic biology and robot isolation with structural analysis all provide promising techniques for discovery on novel natural products [25]. Different from the traditional manual drug experiments with high investment, high risk, low benefits as well as low efficiency, the advanced screening techniques have essential social and economic benefits with more efficiency and low experimental costs [27–29]. The screening of targeted drug is tightly related to bioinformatics, the discovery and validation of new drug cannot be separated from bioinformatics work on molecular target. Using these techniques, research has already screened and discovered potential lead compounds targeting STING, MMP9, EZH2, etc. which all demonstrates good efficacy on different diseases, due to their editability, modifiability and availability [30–32]. Consequently, making full use of pharmacological characteristics of natural products has become a research hotspot in recent years. Based on the current hotspot, a preprint has been previously published by our scientific group in 2021 [33].

Recent researches have gradually focused on the relationships between JAKs and bone-related diseases like OA, OP and IDD [10, 11, 18, 34]. Therefore, this study focused on the chemical structure of JAK3 protein, referred to the molecular interaction mode of FM-381 with JAK3, and combined the techniques of structural biology and high-throughput screening, to discover the potential natural compounds that could effectively dock and inhibit the function of JAK3. A set of high-precision techniques such as high-throughput virtual screening, molecular docking, pharmaceutical/toxicity prediction, molecular dynamics simulation and other structural biology methods were performed and comprehensively evaluated to achieve the goal.

## MATERIALS AND METHODS

### Data collection and acquisition

Bulk RNA-seq dataset GSE42611 was obtained from Gene Expression Omnibus repository (GEO, https://www.ncbi.nlm.nih.gov/geo/) for weighted gene co-expression network analysis (WGCNA) and differentially expressed genes (DEGs) analysis [35]. GSE42611 contained 4 normal and 4 degenerative nucleus pulposus (NP) tissues. In the selection of scRNA-seq data, the following criteria were included: standard surgical protocol was performed to ensure only annulus fibrosis (AF) and NP tissues were isolated by experienced surgeons, and dataset with only NP and AF cells after IVD cells filtration by centrifugation. Consequently, single-cell RNA-seq dataset GSE199866 was downloaded for in-depth analysis based on single-cell resolution, which contained 1 normal NP tissue, 1 degenerative NP tissue, 1 normal annulus fibrosis (AF) tissue and 1 degenerative AF tissue. The samples were prepared by the separation of NP and AF tissues from degenerative and non-degenerative IVD in the same individual [36].

### Weighted gene co-expression network analysis

WGCNA analysis was performed to identify corresponding expression modules. All genes of the NP tissues were from GSE42611, totally 18870 genes were included for network establishment to get the comprehensive results. The outliers from NP tissues were detected and eliminated by hierarchical samples clustering for sample quality control, and the outlier of samples was 0, that meant all samples could be included for WGCNA workflow. Soft threshold power was calculated to construct WGCNA network. After the co-expression network was established, gene patterns in each module were identified to cluster the similar genes together. The correlations between clinical traits (IDD) and the identified module were analyzed to screen the interested module. Eigenvalues of each module as well as the clinical trait (IDD) was also calculated to cluster the similar objects together. Among the above analysis, the most significant module was determined as the interested module for subsequent research.

### Differentially expressed genes identification

The DEGs between degeneration and non-degeneration NP tissues were further analyzed and identified according to the gene expression matrix using “limma” algorithm. The cut-off threshold was set as adjusted P value < 0.05 and log2|fold change (FC)| > 2. Volcano scatter plot was depicted to visualize the distribution of these changed genes.

### Functional and pathways enrichment analysis of DEGs

“ClusterProfiler” R package was conducted for the Gene Ontology (GO), Kyoto Encyclopedia Genes and Genomes (KEGG) analysis based on the analyzed DEGs, to get comprehensive knowledge interpretations about the biological functions as well as aberrantly activated signaling pathways. P < 0.05 was set as the cut-off value. And Gent Set Enrichment Analysis (GSEA) was further performed to get more potential biological functions and pathways to avoid being ignored by GO and KEGG analysis. The annotation gene sets were set as reference standard, and P < 0.05, gene size > 10 were set as the cut-off value.

### Single-cell transcriptome integration and monocle analysis

The quality of cells was detected through a rigorous criterion: cells had fewer than 200 expressed genes and mitochondria UMI counts rate > 5% were eliminated, and cells with less than 5% mitochondrial and 5% erythrocyte were included for subsequent analysis, these cells were considered as eligible cells and included for single-cell integration. The influences of cell cycle-related genes were also eliminated. Then Seurat alignment procedure was conducted to create single-cell object [37]. RunPCA algorithm was conducted to recognize common variation and anchors to integrate different samples and remove the batch effects. An unsupervised nonlinear dimensionality reduction technique was applied for the first 30 PCs, followed by t-distributed stochastic neighbor embedding (tSNE).

Monocle analysis was then performed to analyze the significant changed genes between degeneration and non-degeneration NP tissues, with “tSNE” and default parameters set [38]. Then volcano plot was depicted to visualize the distribution of these significant changed genes.

### PPI network construction and hub genes screening

Based on the above analysis of WGCNA, limma, and single-cell Monocle analysis, Venn plot analysis among the generated DEGs in each part was further conducted to shrink the candidate range, the further screened genes were considered as common DEGs.

This study then performed STRING database (Search Tool for the Retrieval of Interacting Genes, https://string-db.org/) [39], to construct protein-protein interaction (PPI) network of these common DEGs. Then the connected genes were put into Cytoscape software to visualize and generate PPI network from STRING database, to recognize the most related hub genes. “Cytohubba” plug-in was used to calculate the hub genes among these common DEGs, the highest “Degree” value was finally determined as the hub genes of IDD.

### Advanced structural biology approach and natural products repository

The high-throughput virtual screening workflow was clearly reported early in our pre-print version, the detailed information could be seen as previously described [33].

Discovery studio 4.5 software (DS 4.5) is designed and applied for molecular modeling and designing based on macromolecules, which is also applied for drug discovery, interaction analysis as well as functional research [30, 40]. This study applied the natural products in ZINC15 database to screen the potential JAK3 inhibitors. The ZINC15 database is a free database of commercially available compounds for virtual screening, which is provided by the Irwin and Shoichet Laboratories, Department of Pharmaceutical Chemistry, University of California, San Francisco (San Francisco, CA, USA) [41].

### Structure-based virtual screening

The Libdock module was applied for high-throughput virtual screening, which was fast docking module. The chemical structure complex of JAK3 protein was docked with reference drug FM-381, which was retrieved from the protein data bank (https://www.rcsb.org/, PDB ID: 6GLB), with the resolution of 1.9-Å. The protein was prepared by deleting peripheral crystal water and other heteroatoms, followed by ionization, protonation, addition of hydrogen, as well as energy minimization. The active site for docking was referenced with the binding site of the initial ligand (FM-381) position. All these compounds were performed by Libdock at the defined binding site for high-throughput virtual screening. Then all the docked poses were ranked and grouped by Libdock score.

### Predictions of the drugs properties

The ADME module was conducted to predict the absorption, distribution, metabolism, excretion of the candidate compounds, including cytochrome P450 2D6 (CYP2D6) inhibition, aqueous solubility, plasma protein binding (PPB) level, hepatotoxicity, blood-brain barrier (BBB) penetration, as well as human intestinal absorption. TOPKAT (Toxicity Prediction by Komputer Assisted Technology) module was also conducted to predict the toxicity and other properties of these candidate compounds, including developmental toxicity potential (DTP), rodent carcinogenicity (referenced by FDA standards) and Ames mutagenicity. These characteristics were fully evaluated in the selection of potential JAK3 candidate drugs.

### Molecule docking mechanisms analysis

CDOCKER docking method was performed for precise docking analysis based on CHARMm forcefield, which generated accurate docking results. The CHARMm forcefield was applied for both receptors and ligands. Receptor was held rigid, while ligands were allowed to flex in the docking process. The CDOCKER method calculated the interaction energy between each complex and JAK3 protein, which suggested ligand binding affinity. The crystal water molecules of JAK3 were generally deleted, because crystal water molecules may influence the conformation of the complex. After removing water molecules, hydrogen atoms were added to the JAK3 protein. To prove the reliability of reproduction, the inhibitor compound FM-381 was extracted from the initial conformation of FM-381-JAK3 complex, and then FM-381 was redocked into the binding site of JAK3 to compare the root-mean-square deviation (RMSD) with the initial conformation. During the docking process, the ligands were allowed to bind with the residues of protein within the binding site sphere. Different conformations of each ligand-JAK3 complex were generated and visualized to analyze the binding patterns.

### Molecular dynamics simulation

This study selected the most appropriate binding conformation of the ligand-JAK3 complexes among the conformations through CDOCKER module, which were then conducted for molecular dynamics simulation. The CHARMm forcefield was applied to the complex, and solidum chloride were fixed to the process with the ionic strength of 0.145 to simulate the physiological environment in body. Molecular dynamics simulation (production module) was applied for 300 ps with time step of 1 fs. The whole procedure was conducted under normal pressure and temperature. The initial complex was considered as reference, the alterations during the whole process were determined by RMSD, energy values, as well as structural characteristics followed by standard protocol.

### Primary tissues collection and reagents

The primary Rat nucleus pulposus (NP) tissues were collected from animal center of Air Force Medical University. This study protocol was authorized by the Ethics Committee of Xijing Hospital, Air Force Medical University. Tert-butyl hydroperoxide (TBHP) were purchased from Sigma-Aldrich (CAS No. 75-91-2, St. Louis, MO, USA). After primary NP cells were simulated with different concentrations of TBHP (50, 100 μM), this study conducted qRT-PCR to detect the relative expression of the hub genes VEGFA, MMP3, TNFSF11, and TIMP3, respectively, to verify the hallmark genes in the progression of IDD.

### Cell culture and treatment

The primary Rat NP cells were separated by standard protocol, and then cultured in 10% fetal bovine serum (FBS; Invitrogen, Waltham, MA, USA), 1% antibiotics (penicillin/streptomycin) (CAS No. 15140122, Gibco, USA) and DMEM/F12 culture medium (1:1) (CAS No. 12634010, Gibco) at 5% CO_2_ and 37° C conditions [42, 43]. TBHP with different concentrations (50, 100 μM) were added to the complete culture medium to imitate the NP degeneration model. The drug ZINC000072131515 was obtained by Selleck Chemical (CAS No. s5082, USA). Different concentrations of the drug were added to investigate the mitigation effects on cells apoptosis.

### Cell viability assay

The NP cells activity was tested by CCK-8 assay (CAS No. K1018, ApexBio, USA). Cells were plated into 96-well culture plates overnight; each well was controlled by the density of 5000 cells. The TBHP-induced NP cells were processed with different concentrations of ZINC000072131515 for 24 h accordingly; then 10 μL CCK-8 kit was added to each well to measure the signals strength by microplate reader (Synergy H1, USA) to reflect the cell activity.

### Migration capacity assay

The NP cells were seeded into 6-well plate to assess the migration ability by ZINC000072131515 treatment. 1-ml pipette tip was used to make empty area and PBS was used to wash the cell fragments. After addition of ZINC000072131515 into the degenerative NP cells, the images of the empty area were recorded with phase contrast microscopy and the area width was calculated.

### Western blot assay

The NP cells were seeded into T25 culture flask and treated with different concentrations of ZINC000072131515 for 24h. These NP cells were collected and lysed in RIPA buffer containing protease inhibitors. After centrifugation, BCA protein assay kit was used to determine the concentrations of each sample. Each sample was separated in sodium dodecyl sulfate-polyacrylamide gel (SDS-PAGE) and then transferred to polyvinylidene difluoride (PVDF) membranes. After blocked by 5% skim milk (the powder was dissolved in Tris-buffered saline with 0.1% Tween 20) for 2 h at room temperature, the membranes were incubated with primary antibodies against JAK3 and GAPDH (Abcam, Cambridge, United Kingdom) at 4° C overnight. Then the membranes were further mixed with secondary antibody for 1h at room temperature. Last, the generated bands were blended with enhanced chemiluminescence reagents to detect signal strength, Viber Bio Imaging tool was employed to quantify the band signal strength.

### Availability of data and material

The data used and analyzed in this study are available upon reasonable request.

### Consent for publication

All contributing authors have agreed with the publication of this article.

## RESULTS

### Establishment of the WGCNA network

**Figure 1:**
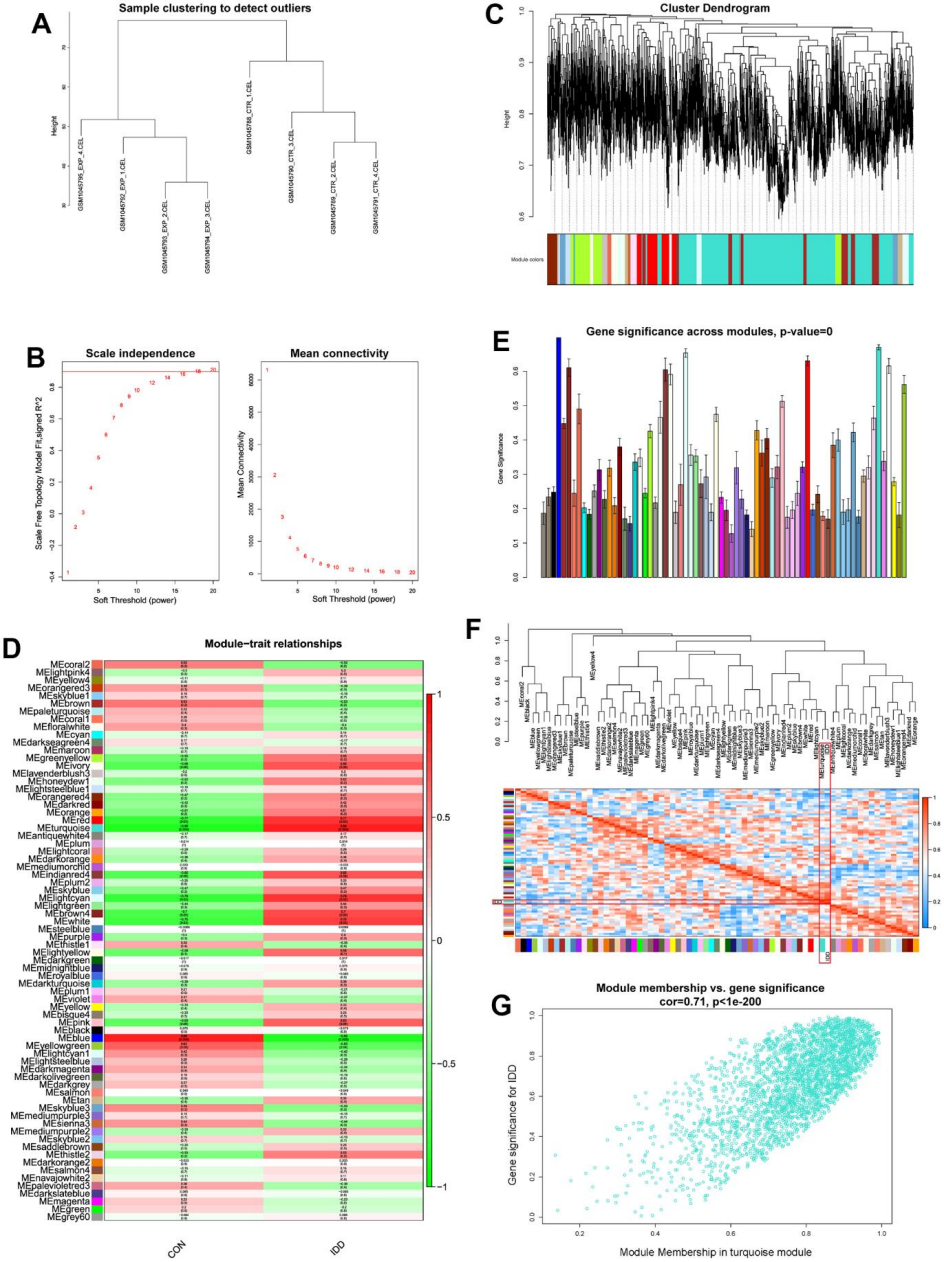
(**A**) Sample clustering analysis to detect the outliers. (**B**) Determination of soft threshold power value. Left panel indicated scale-free model fit index; right panel indicated the mean connectivity of these values. (**C**) Dendrogram branch plot of genes based on dissimilarity measure and assignment modules. (**D**) Module-trait correlation heatmap between different clinical traits and modules. (**E**) Gene significance histogram plot of all clustered modules. (**F**) Eigenvalue correlation heatmap of the modules and clinical traits. (**G**) Correlation scatter plot between gene-significance and module membership.

WGCNA was applied to identify the most correlated relationships between clinical trait and the identified module. All genes were put into the establishment of co-expression network. Quality control by sample clustering analysis did not display outliers, indicating that all samples could be employed for WGCNA workflow (Figure 1A). The soft threshold power value was determined as 16 when R^2^ reach 0.90 and the mean connectivity infinitely approached 0 (Figure 1B). Then these genes were clustered to generate the co-expression network, this study performed hierarchical clustering tree analysis based on the mutual gene expression patterns to cluster the similar genes together. Totally 73 modules were generated with the unique color based on their expression patterns, and dendrogram branch plot illustrated that each module was highly heterogeneous (Figure 1C).

After WGCNA network establishment, this study assessed the relationships between clinical traits (CON and IDD) and different modules. Correlation heatmap visualized those genes in turquoise module had the highest correlations and lowest P values (Cor = 0.88, P = 0.004), elucidating that turquoise module had the highest connections with IDD (Figure 1D). The gene-significance histogram plot validated the conclusion and proved the reliability of turquoise module (Figure 1E). The function of “moduleEigengenes” was used to calculate the detailed expression values of each module, after the expression value of each module was quantified, eigengene adjacency value was then calculated based on the clinical trait and modules. Through the quantize of IDD traits into 0 and 1, the clinical traits were also included for correlations analysis which was then observed by “plotEigengeneNetworks” function. Results demonstrated that turquoise module and phenotype IDD clustered directly (Figure 1F), suggesting that these two eigenvalues interacted tightly with each other. Focusing on the turquoise module, we extracted genes and performed scatter correlations between module and gene-significance, results visualized that these genes significance had significant positive trend in turquoise module (Figure 1G). Therefore, turquoise module was considered as the significant module for the progression of IDD.

### Differentially expressed genes and functional pathways enrichment analysis

**Figure 2:**
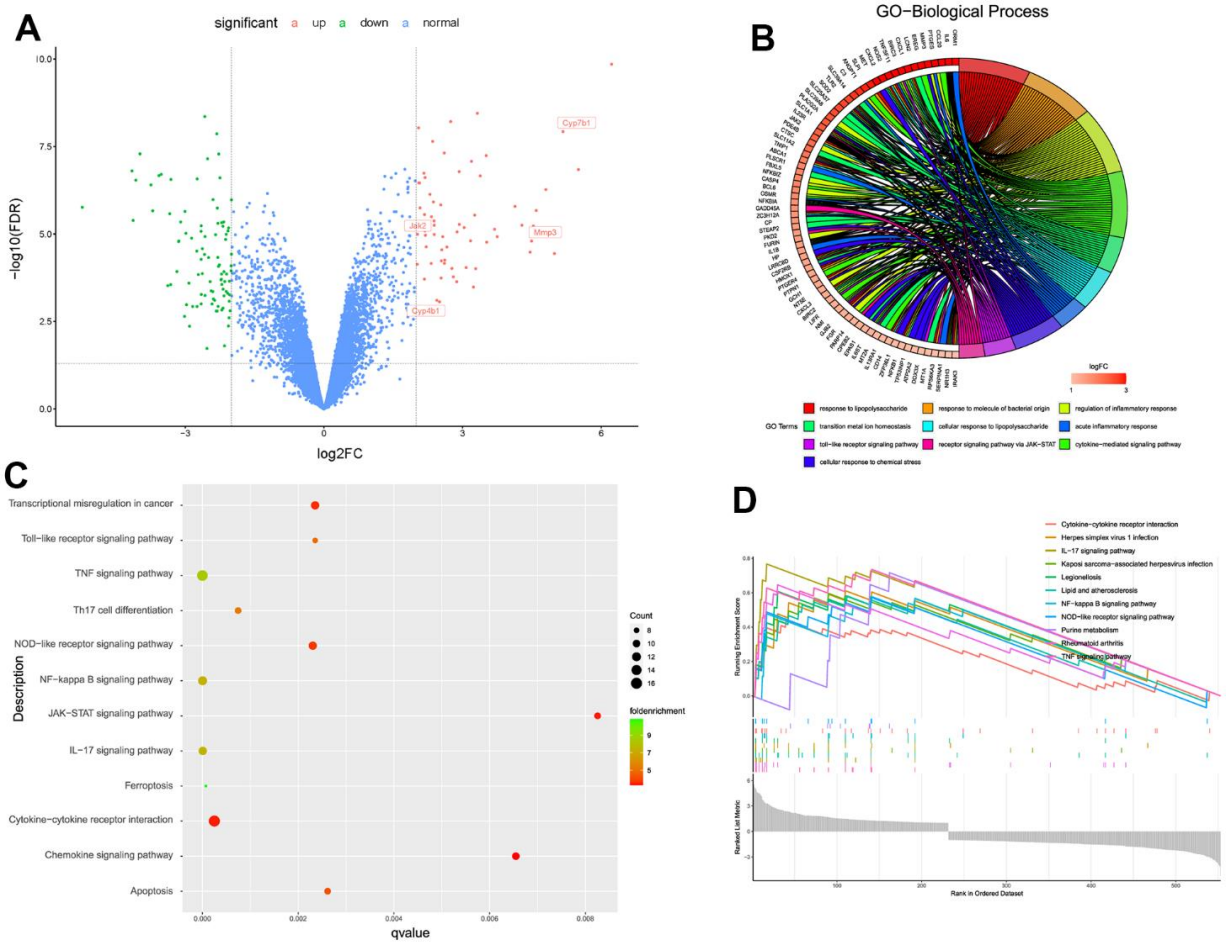
(**A**) Volcano scatter plot of the genes analyzed by limma algorithm. (**B**) Biological functions about the up-regulated DEGs. (**C**, **D**) Aberrantly activated signaling pathways analyzed by KEGG, GSEA, respectively.

This study further used limma algorithm to identify the DEGs from degenerative and non-degenerative NP tissues based on bulk RNA-seq. With the cut-off threshold setting as adjusted P < 0.05 and log2|FC| > 2, totally 657 DEGs were identified, including 270 up-regulated and 387 down-regulated genes, volcano scatter plot visualized the gene distributions about these changed genes (Figure 2A).

Then we conducted biological functions and aberrantly pathways analysis based on the up-regulated DEGs, for further knowledge information about the pathological processes in the progression of IDD. As shown in Figure 2B, results illustrated that the up-regulated genes mainly participated in inflammatory-related responses like “inflammatory response”, “cytokine-mediated response” and “cellular response to lipopolysaccharide” etc. And results also analyzed several aberrantly activated signaling pathways such as TNF, NF-κB, JAK-STAT, IL-related, and apoptosis signaling pathways (Figure 2C, 2D). These biological functions and pathways exhibited valuable roles, that they both may regulate the development of IDD, which were worth further discovering the potential natural materials based on the specific signaling pathway.

### Single-cell transcriptome integration and monocle analysis

To get more accurate results based on single-cell resolution, this study obtained NP tissues from the same individual to avoid differences like age, gender and other indicators etc. After the rigorous quality control about these cells, totally 7601 cells were existed in NP tissues, with 3665 cells in degenerative sample and 3936 cells in non-degenerative sample, together with 19941 expressed genes (Supplementary Figure 1A). The number of genes in this study was positively correlated with the sequencing depth, and mitochondria or erythrocyte UMI rate were all lower than 5% (Supplementary Figure 1B). After integration analysis by RunPCA algorithm, the plots did not show distinct separations in the scatter plot, indicating good integration results (Supplementary Figure 1C). Furthermore, this study also eliminated the effects of cell cycle-related genes (Supplementary Figure 1D).

**Figure 3:**
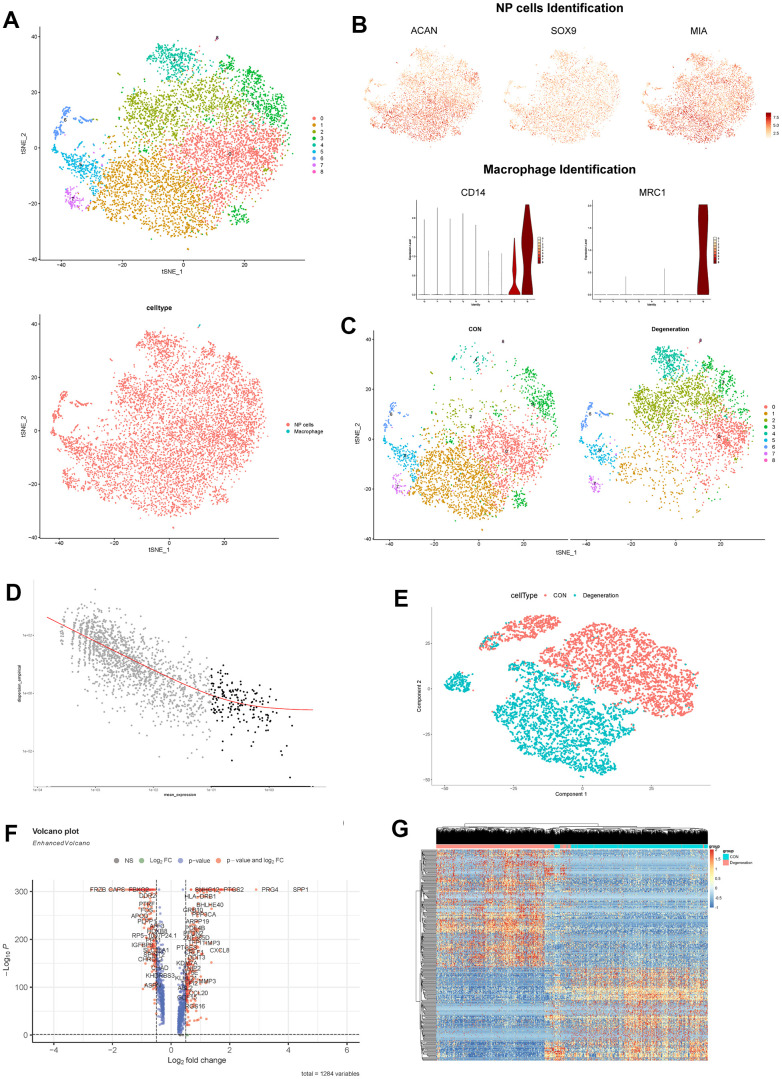
(**A**) tSNE visualization of all cells in NP tissues after sample integration, 9 clustered were generated. (**B**) Dot plot showing the NP cells marker genes ACAN, SOX9 and MIA within tSNE map; and violin plot showing the expression of macrophage marker genes CD14 and MRC1. (**C**) tSNE visualization of all cells in NP tissues between degeneration and non-degeneration group. (**D**) Selection of the most variable genes for monocle analysis. (**E**) PCA dimensional reduction by monocle algorithm between degeneration and non-degeneration group. (**F**) Volcano scatter plot of the genes analyzed by monocle method. (**G**) Hierarchical clustering heatmap of the analyzed DEGs in each cell.

After dimensional reduction method by “tSNE” algorithm, the single-cell Seurat object generated 9 distinct clusters (Figure 3A), which were annotated for 2 main cell populations, including macrophages and NP cells. The NP cells were annotated by markers of SOX9, ACAN, and COL2A1 (clusters 0-7) [44, 45]; and macrophage cells were annotated by markers of CD14 and MRC1 (cluster 8) [46], as shown in Figure 3B.

Through the tSNE plot between non-degeneration and degeneration NP tissues, we observed distinct cell distribution (Figure 3C), that meant the cell proportion and transcriptome were changed between different states. Then this study performed monocle analysis to detect the significant changed genes between non-degeneration and degeneration NP tissues. After noise filtering, the most variable genes were selected and employed for monocle analysis (Figure 3D). Monocle algorithm could sort and cluster different cells through machine learning technique (reversed graph embedding), which could solve the complex biological process accurately and reliably. Monocle analysis was mainly used to identify the DEGs at different stages and in different cell types [38]. Results displayed that after monocle dimensional reduction, the cells between non-degeneration and degeneration NP tissues had highly heterogeneity, different groups could be distinguished significantly by those genes (Figure 3E). Monocle differential analysis analyzed that those 288 genes had significant roles in mediating different states of NP tissues (Figure 3F). Cells heatmap further visualized the gene expression profiles in each cell (Figure 3G).

### PPI network construction and hub genes screening

**Figure 4:**
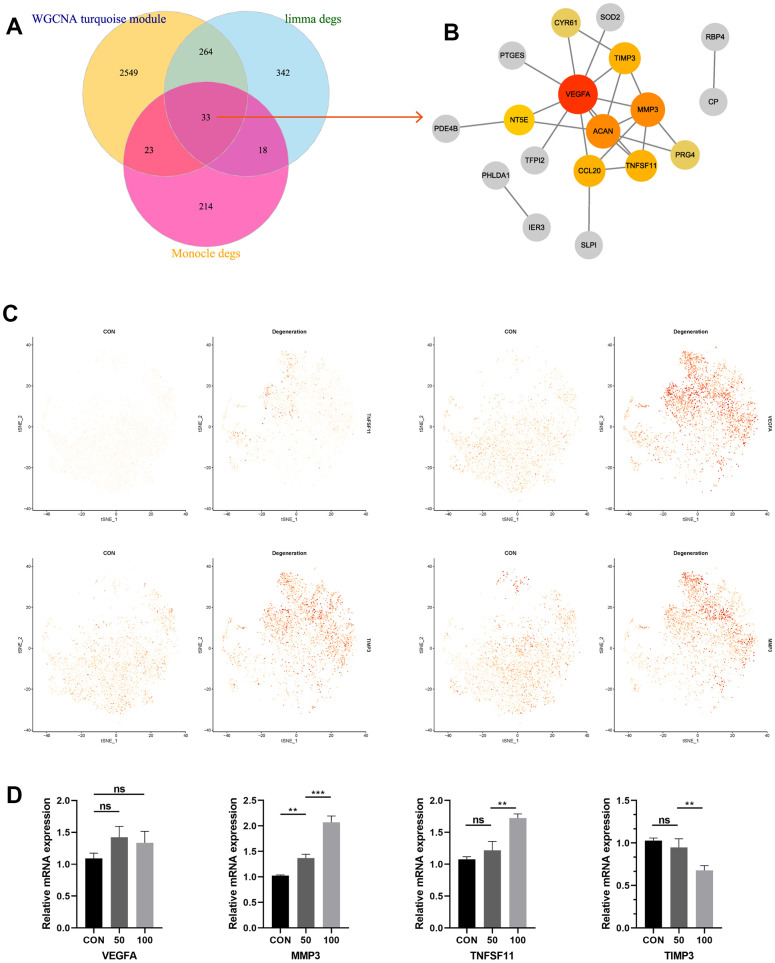
(**A**) Venn diagram suggesting 33 genes were commonly expressed genes in three parts. (**B**) PPI construction of the commonly expressed genes to identify the most significant hub genes. (**C**) Featureplot visualization about the hub genes between degeneration and non-degeneration group at single-cell resolution. (**D**) Relative mRNA expression levels of the four hub genes in the control and degeneration groups.

Based on the results about turquoise module from WGCNA analysis, DEGs from limma analysis, as well as monocle results from single-cell RNA analysis, this study employed Venn plot analysis to shrink the range of the candidate genes, and totally 33 genes were considered as common DEGs (Figure 4A). Then we constructed PPI network from STRING database to identify the most related genes. Through “Cytohubba” algorithm calculation, the hub genes were finally determined by the highest “degree” values, namely VEGFA, MMP3, TNFSF11, and TIMP3, respectively (Figure 4B). Single-cell Featureplot further visualized the gene expression distributions in each cell between non-degeneration and degeneration NP tissues (Figure 4C). Furthermore, the relative expressions of these hallmark genes were also detected by qRT-PCR analysis, results demonstrated that compared to the control group, the expressions of MMP3, TNFSF11 were significantly elevated in degeneration group, and the expression of TIMP3 was reduced in degeneration group (p < 0.05). However, the expression levels of VEGFA did not reach the expected results of the experiments (Figure 4D). We hypothesized the reason was that through the former analysis, the NP tissues were collected from the IVD tissue, that meant other cell types could also be collected as bulk, such as macrophages, while this study used cell lines to validate the expression. VEGFA were generally expressed in macrophages and other cell types like vascular cells, etc., consequently, the VEGFA discovered in this study was more likely to exist in other cell types, nor NP cell lines. That’s the reason why the expression of VEGFA in qRT-PCR analysis was far from satisfactory in bulk RNA analysis.

Through the KEGG analysis based on all the identified DEGs, we observed that JAK-STAT signaling pathway was significantly activated, as shown in Figure 2C. The generated signaling pathways obtained by including all DEGs were often more convincing, since the results were integrated by diversity genes. Besides, the JAK-STAT signaling pathway was reported to be closely connected with the progression of IDD [10, 11]. Therefore, this study further focused on JAKs protein to perform the following high-throughput virtual screening.

### High throughput virtual screening against JAK3

**Figure 5:**
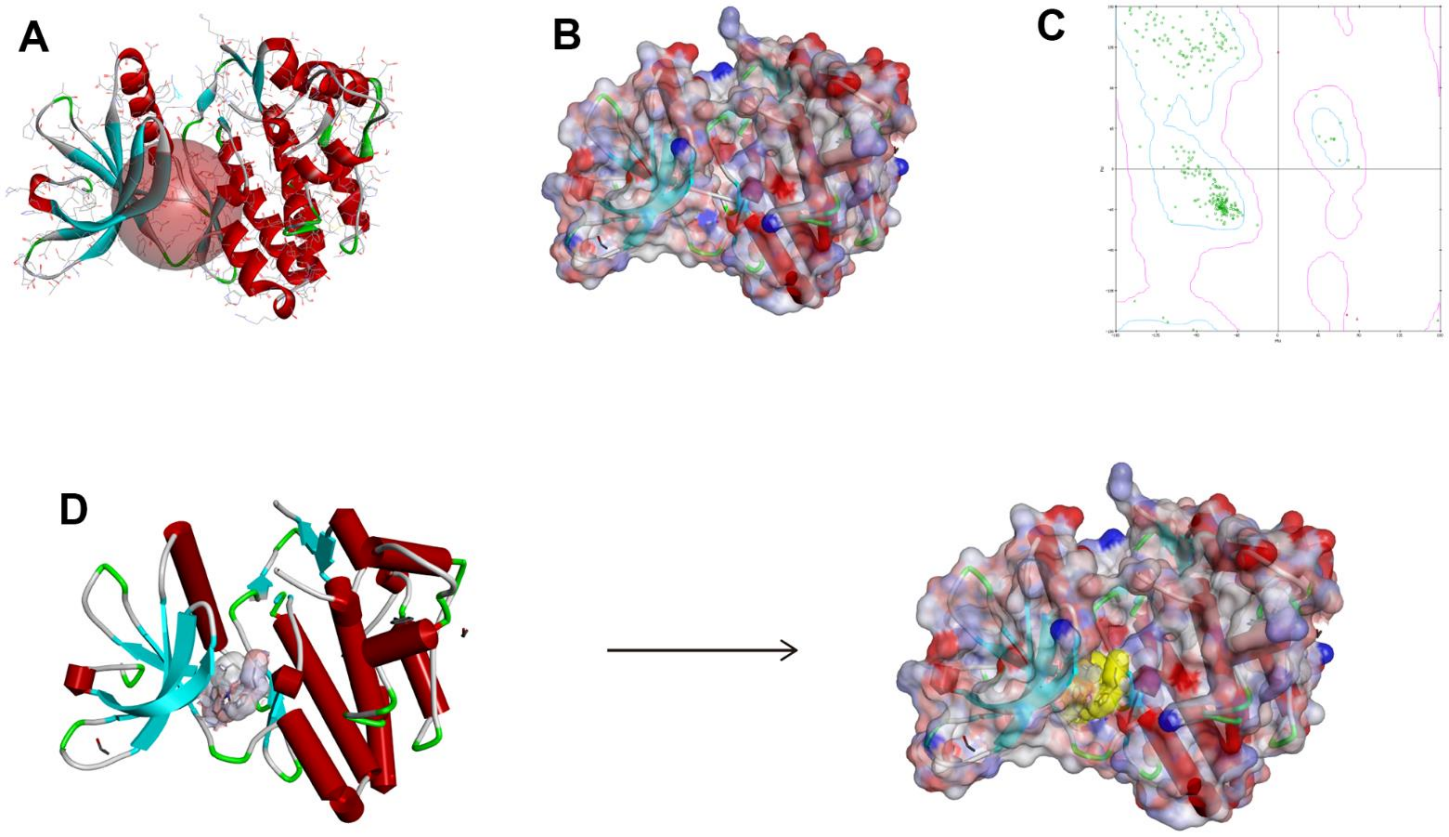
**The chemical structure of Janus kinase 3 (JAK3).** (**A**) Initial crystal structure of JAK3 with active binding sphere addition. (**B**) Macromolecule with surface binding region surrounded, red indicated positive charge and blue indicated negative charge. (**C**) The Ramachandran chart of JAK3 macromolecule. (**D**) Docking structure model of FM-381-JAK3 complex.

**Figure 6:**
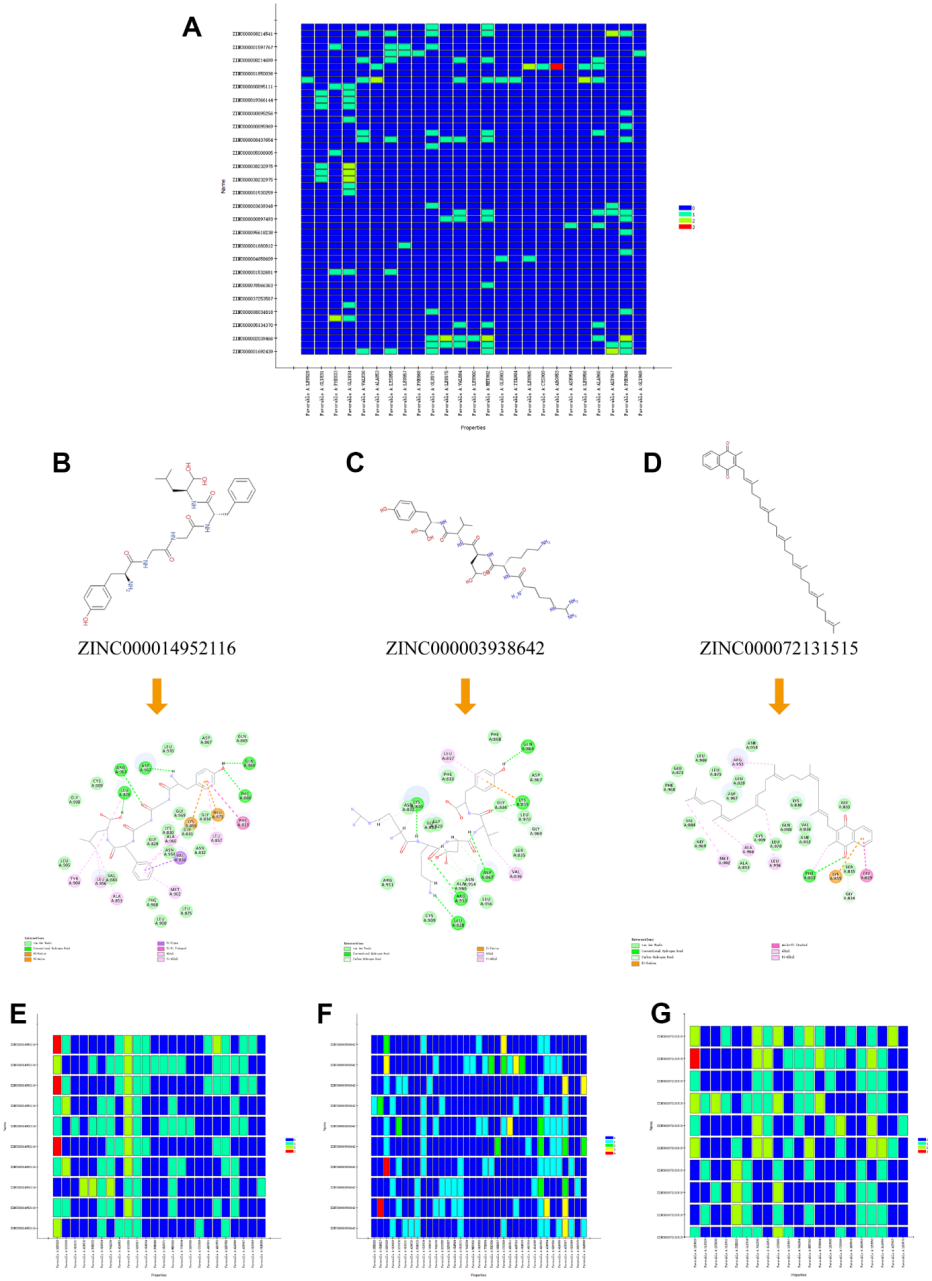
(**A**) Residues interaction roles heatmap based on the top 50 compounds from Libdock module. (**B**–**D**) Detailed intermolecular interactions between ZINC000014952116-JAK3, ZINC000003938642-JAK3 and ZINC000072131515-JAK3, respectively. (**E**–**G**) Residues interaction roles heatmap of ZINC000014952116-JAK3, ZINC000003938642-JAK3, and ZINC000072131515-JAK3 complex.

Small molecules binding to arginine residues region may inhibit the active sites of JAK3 and thereby suppressing its functions, which was an essential regulatory site of JAK3 and behaved pivotal roles in regulating its function. The chemical structure and Ramachandran information of JAK3, as well as docking conformation of FM-381-JAK3 complex were illustrated in Figure 5A–5D. After high-throughput screening method from amounts of prepared ligands based on Libdock module, altogether 13653 compounds from ZINC15 database bound successfully at the active region of JAK3 protein, 1028 compounds had higher Libdock scores (range from 127.612 to 212.521) than the reference compound (Libdock score: 127.568). The Libdock scores of the top 50 compounds were shown in Table 1, which were screened as candidate compounds. Besides, the heatmap displayed the interactive roles between ligand residues of the top 50 compounds and JAK3 (Figure 6A). The chemical structures of the top 20 compounds were also illustrated in Supplementary Figure 2.

**Table 1:** Top 50 ranked compounds with higher Libdock scores than FM-381 drug.

**Number**	**ZINC ID**	**Libdock score**	**Number**	**ZINC ID**	**Libdock score**
1	ZINC000042805482	212.521	26	ZINC000049784088	176.625
2	ZINC000085545908	195.857	27	ZINC000095620579	176.612
3	ZINC000062238222	195.269	28	ZINC000003830635	176.014
4	ZINC000085541163	194.48	29	ZINC000014233122	175.406
5	ZINC000095620524	192.712	30	ZINC000014712793	175.244
6	ZINC000150338786	192.491	31	ZINC000003995616	175.076
7	ZINC000085826837	191.853	32	ZINC000004096684	174.649
8	ZINC000004096878	189.333	33	ZINC000008552069	174.507
9	ZINC000085544839	185.794	34	ZINC000004096653	174.313
10	ZINC000003938642	185.518	35	ZINC000026671872	174.266
11	ZINC000085826835	183.589	36	ZINC000004097774	174.25
12	ZINC000004099069	182.369	37	ZINC000095617636	174.108
13	ZINC000004096892	181.982	38	ZINC000100045922	173.686
14	ZINC000028968101	181.434	39	ZINC000004654845	173.662
15	ZINC000008220036	180.726	40	ZINC000100822590	173.49
16	ZINC000004099068	180.395	41	ZINC000056897657	173.465
17	ZINC000004096889	179.921	42	ZINC000072131515	173.214
18	ZINC000004096059	179.877	43	ZINC000038143594	172.993
19	ZINC000014952116	179.566	44	ZINC000004096877	172.592
20	ZINC000014946303	179.017	45	ZINC000049878197	172.478
21	ZINC000004217536	178.768	46	ZINC000013513540	171.806
22	ZINC000095617635	178.012	47	ZINC000100277550	171.738
23	ZINC000008220033	177.586	48	ZINC000014951658	171.734
24	ZINC000028968107	177.534	49	ZINC000003933041	171.291
25	ZINC000049878510	177.174	50	ZINC000004096888	171.232
FM-381 (reference compound)					127.568

### Pharmacological properties and toxicity prediction

The ADME algorithm was conducted to predict the drug pharmacological characteristics of these candidate compounds and the reference compound FM-381. Results indicated that the aqueous solubility of these selected ligands varied a wide range, which were the same as the BBB level. As for the reference compound (FM-381) property, it was predicted to be non-inhibition to CYP2D6 and had a high binding force with plasma protein; however, it was predicted to be toxic to liver. The detailed information of pharmacological properties was listed in Table 2.

**Table 2:** Pharmacological properties of the top 20 compounds based on ADME.

**Number**	**ZINC ID**	**Solubility level^a^**	**BBB level^b^**	**CYP2D6^c^**	**Hepatotoxicity^d^**	**Absorption level^e^**	**PPB prediction^f^**
1	ZINC000042805482	2	4	0	0	2	0
2	ZINC000085545908	4	4	0	0	3	0
3	ZINC000062238222	3	4	0	1	3	0
4	ZINC000085541163	2	4	0	0	2	0
5	ZINC000095620524	4	4	0	1	3	0
6	ZINC000150338786	1	4	0	1	3	1
7	ZINC000085826837	2	4	0	0	2	0
8	ZINC000004096878	1	4	0	1	3	1
9	ZINC000085544839	3	4	0	1	3	0
10	ZINC000003938642	0	4	0	0	3	0
11	ZINC000085826835	2	4	0	0	2	0
12	ZINC000004099069	3	4	0	0	3	0
13	ZINC000004096892	2	4	0	1	3	0
14	ZINC000028968101	1	4	1	1	3	1
15	ZINC000008220036	0	4	0	0	3	1
16	ZINC000004099068	3	4	0	0	3	0
17	ZINC000004096889	2	4	0	1	3	0
18	ZINC000004096059	1	4	0	1	3	1
19	ZINC000014952116	4	4	0	0	3	0
20	ZINC000014946303	1	4	0	0	3	0
21	ZINC000004217536	3	4	0	0	3	0
22	ZINC000095617635	2	4	0	0	3	1
23	ZINC000008220033	0	4	0	0	3	1
24	ZINC000028968107	1	4	1	1	3	1
25	ZINC000049878510	1	4	0	0	3	0
26	ZINC000049784088	4	4	0	0	3	0
27	ZINC000095620579	1	4	0	0	3	0
28	ZINC000003830635	5	4	0	0	3	0
29	ZINC000014233122	4	4	0	0	3	0
30	ZINC000014712793	4	4	0	0	3	0
31	ZINC000003995616	1	4	0	0	2	1
32	ZINC000004096684	1	4	0	0	3	1
33	ZINC000008552069	4	4	0	1	3	0
34	ZINC000004096653	1	4	0	0	3	1
35	ZINC000026671872	1	4	0	1	3	1
36	ZINC000004097774	2	4	0	0	3	0
37	ZINC000095617636	2	4	0	0	3	1
38	ZINC000100045922	0	4	0	0	3	1
39	ZINC000004654845	1	4	1	0	3	1
40	ZINC000100822590	1	4	0	1	3	0
41	ZINC000056897657	1	4	0	1	3	0
42	ZINC000072131515	0	4	0	0	3	1
43	ZINC000038143594	3	4	0	0	3	0
44	ZINC000004096877	1	4	0	1	3	1
45	ZINC000049878197	1	4	0	0	3	0
46	ZINC000013513540	4	4	0	1	3	0
47	ZINC000100277550	0	4	0	1	3	1
48	ZINC000014951658	3	4	0	0	3	0
49	ZINC000003933041	1	4	0	0	2	1
50	ZINC000004096888	2	4	0	1	3	0

ADMET (Adsorption, Distribution, Metabolism and Excretion) properties of compounds. ^a^Aqueous-solubility level: 0 (extremely low); 1 (very low, but possible); 2 (low); 3 (good); ^b^Blood Brain Barrier level: 0 (Very high penetrant); 1 (High); 2 (Medium); 3 (Low); 4 (Undefined); ^c^Cytochrome P450 2D6 level: 0 (Non-inhibitor); 1 (Inhibitor); ^d^Hepatotoxicity: 0 (Nontoxic); 1 (Toxic); ^e^Human-intestinal absorption level: 0 (good); 1 (moderate); 2 (poor); 3 (very poor); ^f^Plasma Protein Binding: 0 (Absorbent weak); 1 (Absorbent strong).

Safety and toxicity characteristics also need to be fully evaluated in filtering candidate compounds. This study conducted TOPKAT module to predict the safety of the top 50 compounds by analyzing different kinds of toxic indicators, including Ames mutagenicity, rodent carcinogenicity as well as DTP. The detailed toxic parameters of these candidate drugs were exhibited in Table 3. In terms of the reference compound, it was predicted to have carcinogenicity, together with no DTP potential. Considering the pharmacological properties, ZINC000014952116, ZINC000003938642, ZINC000042805482, ZINC000028968101, ZINC000072131515 and ZINC000008220036 etc., were screened as candidate compounds for the following analysis, owing to their less Ames mutagenicity, rodent carcinogenicity as well as DTP potential, non-CYP2D6 inhibition, not toxic to liver, high solubility in water as well as high intestinal absorption characteristics than other compounds.

**Table 3:** Toxicities properties of the top 50 compounds.

**Number**	**ZINC ID**	**Rat NTP^a^**		**Mouse NTP^a^**		**Ames^b^**	**DTP^c^**
		**Male**	**Female**	**Male**	**Female**		
1	ZINC000042805482	0.998	1.000	1.000	0.186	0.000	1.000
2	ZINC000085545908	0.000	0.000	0.000	1.000	0.000	1.000
3	ZINC000062238222	0.969	0.000	0.000	0.000	0.989	1.000
4	ZINC000085541163	0.969	0.000	0.000	0.000	0.989	1.000
5	ZINC000095620524	0.999	1.000	0.000	1.000	0.000	1.000
6	ZINC000150338786	0.000	0.000	1.000	0.000	1.000	1.000
7	ZINC000085826837	0.998	1.000	1.000	0.186	0.000	1.000
8	ZINC000004096878	0.000	0.000	1.000	0.000	1.000	1.000
9	ZINC000085544839	0.964	0.000	0.000	0.000	0.999	1.000
10	ZINC000003938642	0.000	0.000	0.000	0.000	0.000	0.000
11	ZINC000085826835	0.998	1.000	1.000	0.186	0.000	1.000
12	ZINC000004099069	0.000	0.000	0.000	0.000	0.002	0.864
13	ZINC000004096892	0.000	0.000	0.000	1.000	1.000	1.000
14	ZINC000028968101	0.997	0.060	0.021	1.000	1.000	1.000
15	ZINC000008220036	0.000	1.000	1.000	0.000	1.000	0.000
16	ZINC000004099068	0.000	1.000	1.000	0.000	1.000	0.000
17	ZINC000004096889	0.000	0.000	0.000	0.855	1.000	1.000
18	ZINC000004096059	1.000	0.000	0.000	0.000	0.000	1.000
19	ZINC000014952116	0.000	0.000	0.000	0.001	0.000	0.928
20	ZINC000014946303	0.000	0.000	1.000	1.000	0.000	0.992
21	ZINC000004217536	0.000	0.000	0.000	0.000	0.000	1.000
22	ZINC000095617635	0.002	0.242	0.999	0.000	0.019	0.000
23	ZINC000008220033	0.000	1.000	1.000	0.000	0.000	0.000
24	ZINC000028968107	0.997	0.060	0.021	1.000	1.000	1.000
25	ZINC000049878510	1.000	0.000	1.000	0.816	0.000	0.908
26	ZINC000049784088	0.008	0.000	0.000	0.995	1.000	1.000
27	ZINC000095620579	1.000	0.000	0.000	0.000	0.001	0.000
28	ZINC000003830635	0.999	1.000	0.940	0.004	0.998	0.000
29	ZINC000014233122	0.017	0.000	0.000	0.422	1.000	1.000
30	ZINC000014712793	0.632	1.000	0.000	0.640	0.000	1.000
31	ZINC000003995616	0.000	0.003	0.000	0.003	0.000	0.937
32	ZINC000004096684	0.000	1.000	1.000	0.000	1.000	1.000
33	ZINC000008552069	0.997	0.000	0.001	0.031	0.024	1.000
34	ZINC000004096653	0.000	1.000	1.000	0.000	1.000	1.000
35	ZINC000026671872	0.000	0.000	0.000	1.000	1.000	0.073
36	ZINC000004097774	0.053	0.987	0.916	0.000	0.002	1.000
37	ZINC000095617636	0.002	0.242	0.999	0.000	0.019	0.000
38	ZINC000100045922	0.000	0.000	0.000	1.000	1.000	0.000
39	ZINC000004654845	0.000	1.000	1.000	0.000	1.000	1.000
40	ZINC000100822590	1.000	0.000	0.000	0.000	0.000	1.000
41	ZINC000056897657	0.000	1.000	0.982	0.000	1.000	1.000
42	ZINC000072131515	0.000	1.000	1.000	0.000	1.000	1.000
43	ZINC000038143594	0.088	0.274	0.000	0.061	0.000	1.000
44	ZINC000004096877	1.000	0.000	0.006	0.000	0.000	1.000
45	ZINC000049878197	1.000	0.000	1.000	0.819	0.000	0.908
46	ZINC000013513540	0.840	0.020	0.000	0.139	0.000	1.000
47	ZINC000100277550	0.000	0.000	0.077	0.016	1.000	0.835
48	ZINC000014951658	0.000	1.000	0.000	1.000	0.000	1.000
49	ZINC000003933041	0.362	1.000	1.000	0.000	1.000	0.879
50	ZINC000004096888	0.000	0.000	0.000	0.855	1.000	1.000

^a^<0.3 (Non-Carcinogen); >0.8 (Carcinogen); ^b^<0.3 (Non-Mutagen); >0.8 (Mutagen); ^c^<0.3 (Non-Toxic); >0.8 (Toxic).

### Precise docking patterns of the candidate compounds

**Figure 7:**
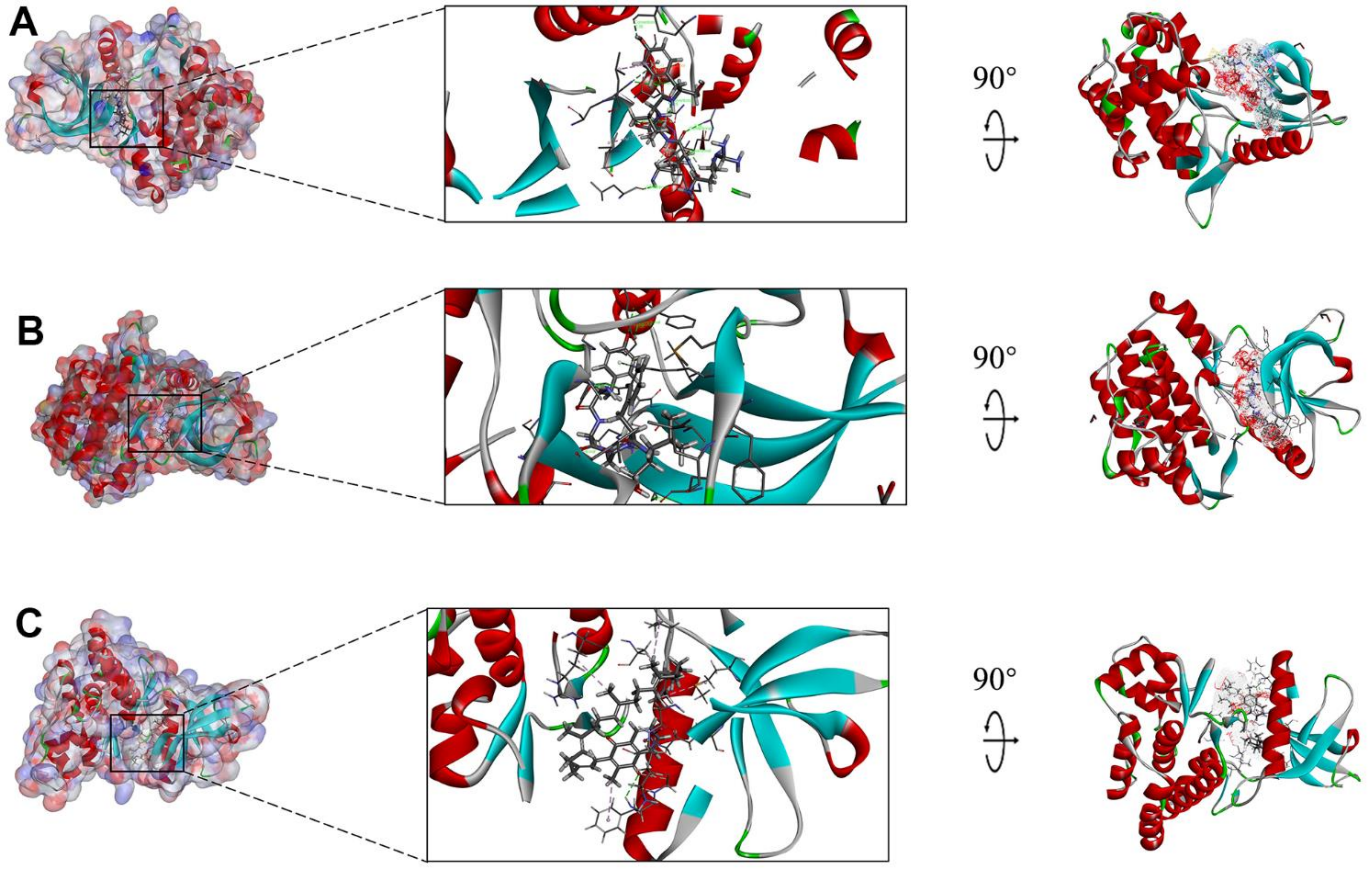
**Schematic drawing of interactions between ligands and JAK3, the surface of binding area was added, blue represented positive charge, red represented negative charge, and ligands were shown in sticks, the structure around the ligand-receptor junction was shown in thinner sticks.** (**A**) ZINC000014952116-JAK3 complex, (**B**) ZINC000003938642-JAK3, and (**C**) ZINC000072131515-JAK3 complex.

The interactions analysis between these candidate compounds and JAK3 was conducted by CDOCKER module to understand the interactive patterns, which is an accurate docking algorithm to generate more reliable results. RMSD between the docked conformation and the initial conformation of the FM-381-JAK3 complex was 0.7413Å, indicating that the CDOCKER method was credible for reproducing the results. After docking the candidate compounds into the binding sphere of JAK3, results suggested that the binding affinity of ZINC000003938642 (-51.5194 kcal/mol) and ZINC000014952116 (-54.1495 kcal/mol) with the protein was lower than FM-381-JAK3 complex (-49.2387 kcal/mol), as listed in Table 4. Structural chemical bonds analysis was applied to calculate different chemical bond interactions. As shown in Figure 6B–6D, results displayed that JAK3 generated 13 pairs of hydrogen bonds, 1 pair of alkyl bonds, 1 pair of π-π interactions, and 1 pair of π-alkyl interactions with ZINC000003938642. JAK3 formed 4 pairs of hydrogen bonds, 2 π-sigma interactions, and 2 π-alkyl interactions with ZINC000014952116. JAK3 generated 2 pairs of hydrogen bonds, 5 pairs of π-alkyl interactions, and 1 π-cation interactions with ZINC000072131515. As for the reference compound FM-381, it formed 7 pairs of hydrogen bonds, 8 π-related interactions, and 2 alkyl interactions. The detailed information of the hydrogen bonds and bonding distance between these compounds and JAK3 was listed in Table 5. Figure 6E–6G displayed the possible interactive roles of residues between these compounds and JAK3. The docking conformation and modes of these candidate compounds with JAK3 were visualized in Figure 7B, 7C to compare the interaction modes of the reference compound with JAK3 (Figure 7A). Thus, these three compounds were regarded as potential lead compounds and pooled for subsequent research. The chemical names of ZINC000014952116, ZINC000003938642 and ZINC000072131515 were Enkephalin, Thymopentin and Menaquinone, respectively.

**Table 4:** CDOCKER interaction energy of the candidate compounds with JAK3.

**ZINC ID**	**CDOCKER interaction energy(kcal/mol)**
ZINC000014952116	-54.1495
ZINC000003938642	-51.5194
FM-381	-49.2387

**Table 5:** Hydrogen bond interaction parameters for potential compounds with JAK3 residues.

**Receptor**	**ZINC ID**	**Donor atom**	**Receptor atom**	**Distances (Å)**
JAK3	ZINC000003938642	LYS855:NZ	ZINC000003938642:O35	3.22
		ARG953:NH2	ZINC000003938642:O15	2.94
		ASP967:OD2	ZINC000003938642:H57	3.05
		LEU828:O	ZINC000003938642:H74	2.60
		LYS830:O	ZINC000003938642:H76	2.09
		GLN864:O	ZINC000003938642:H98	2.36
		ASN954:CA	ZINC000003938642:O11	3.19
		GLY969:CA	ZINC000003938642:O35	3.60
		ASP967:OD2	ZINC000003938642:H58	2.81
		ASN954:OD1	ZINC000003938642:H61	2.70
		ARG953:O	ZINC000003938642:H73	2.72
		LYS830:O	ZINC000003938642:H77	2.20
		LYS830:O	ZINC000003938642:H84	2.27
	ZINC000014952116	A:PHE868:N	ZINC000014952116:O35	3.30
		A:ARG953:NH2	ZINC000014952116:O23	3.00
		A:ASP967:OD1	ZINC000014952116:H69	1.97
		A:GLN864:O	ZINC000014952116:H74	2.06
		A:LEU828:O	ZINC000014952116:H78	1.86
		A:LYS855:NZ	ZINC000014952116	3.89
	ZINC000072131515	A:PHE833:HN	ZINC000072131515:O35	3.08
		A:GLY834:HA2	ZINC000072131515:O35	2.96
		A:LYS855:NZ	ZINC000072131515	4.19
		A:GLY829:C,O;LYS830:N	ZINC000072131515	4.65
		A:ALA966	ZINC000072131515:C8	3.34
		A:MET902	ZINC000072131515:C3	5.28
		A:LEU956	ZINC000072131515:C13	5.35
		A:ARG953	ZINC000072131515:C18	4.32
		A:PHE833	ZINC000072131515:C33	4.30

**Figure 8:**
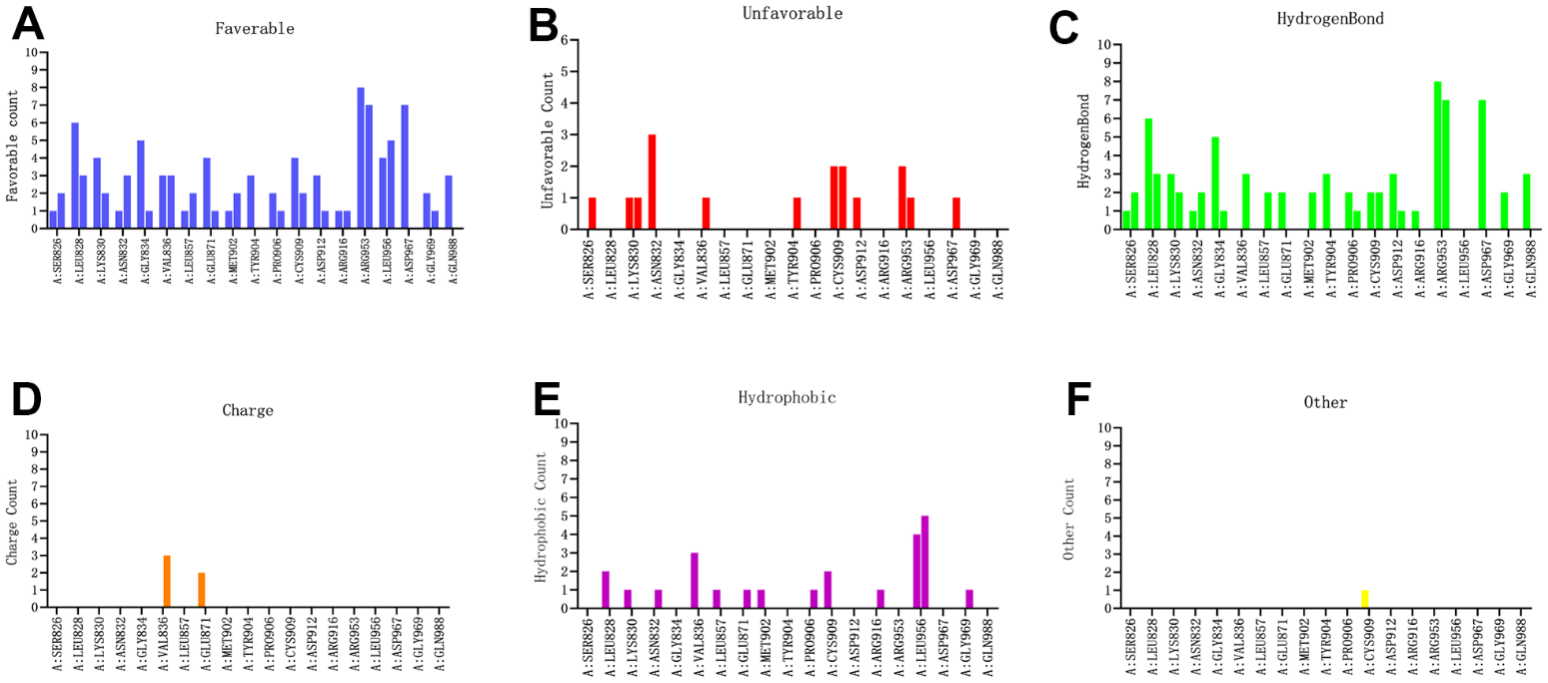
Probability of interactive residues roles between the compounds and JAK3 based on (**A**) favorable count, (**B**) unfavorable count, (**C**) hydrogen bond count, (**D**) charge count, (**E**) hydrophobic count and (**F**) other count.

To further observe the detailed residues alteration information during docking process between the candidate compounds and JAK3, this study calculated that there were favorable counts, unfavorable counts, hydrogen bond counts and hydrophobic counts, respectively. As shown in Figure 8, results illustrated that during docking process, most of the favorable counts, hydrogen bond counts and hydrophobic counts participated in the site docking, while unfavorable counts rarely contributed to the docking complex, indicating that these residues all prompted the stability of ZINC000072131515-, ZINC000014952116- and ZINC000003938642-JAK3 complex.

### *In-vivo* drugs stability evaluation by molecular dynamics simulation

**Figure 9:**
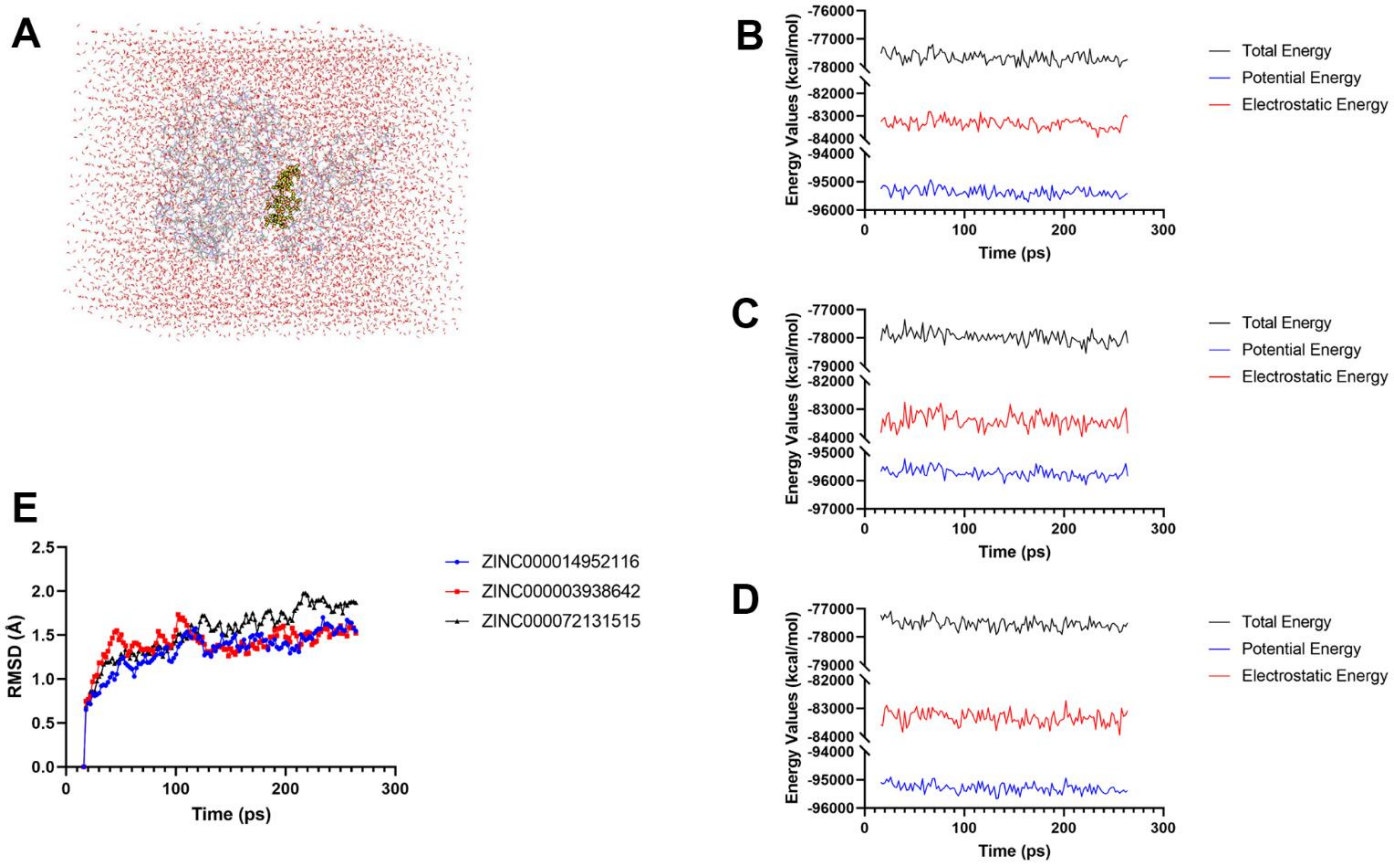
**Results of molecular dynamics simulation of these two compounds.** (**A**) Orthorhombic box with an explicit periodic boundary solvation water model. (**B**–**D**) Energy values of ZINC000014952116-JAK3, ZINC000003938642-JAK3 complex, and ZINC000072131515-JAK3 complex. (**E**) RMSD values of these three compounds.

After analyzing the docking patterns, the stabilities of these generated complexes under *in-vivo* situations were then assessed by molecular dynamics simulation experiments (Figure 9A). RMSD and energy values curves of each complex were illustrated based on the initial conformations through the CDOCKER module. Energy values of these three complexes including total energy, potential energy as well as electrostatic energy began to level off with time (Figure 9B, 9C); the RMSDcurves of these complexes attained equilibrium after 130 ps (Figure 9D). The above results demonstrated that these chemical bonds between compounds and JAK3 help to maintain the homeostasis of these complex.

### The effects of TBHP on the degeneration NP model

TBHP is a stable form of hydrogen peroxide, and is widely applied for constructing degeneration NP model [47]. To evaluate the effects of different concentrations of TBHP on the activities of NP cells, the viability was examined by CCK-8 assay after addition of TBHP for 24h. The NP cells displayed decreased vitality with dose-dependent, and 100μM was chosen for *in-vitro* NP cells degeneration model (Figure 9F).

### ZINC000072131515 ameliorated apoptosis of degenerative NP cells by reducing the JAKs family

**Figure 10:**
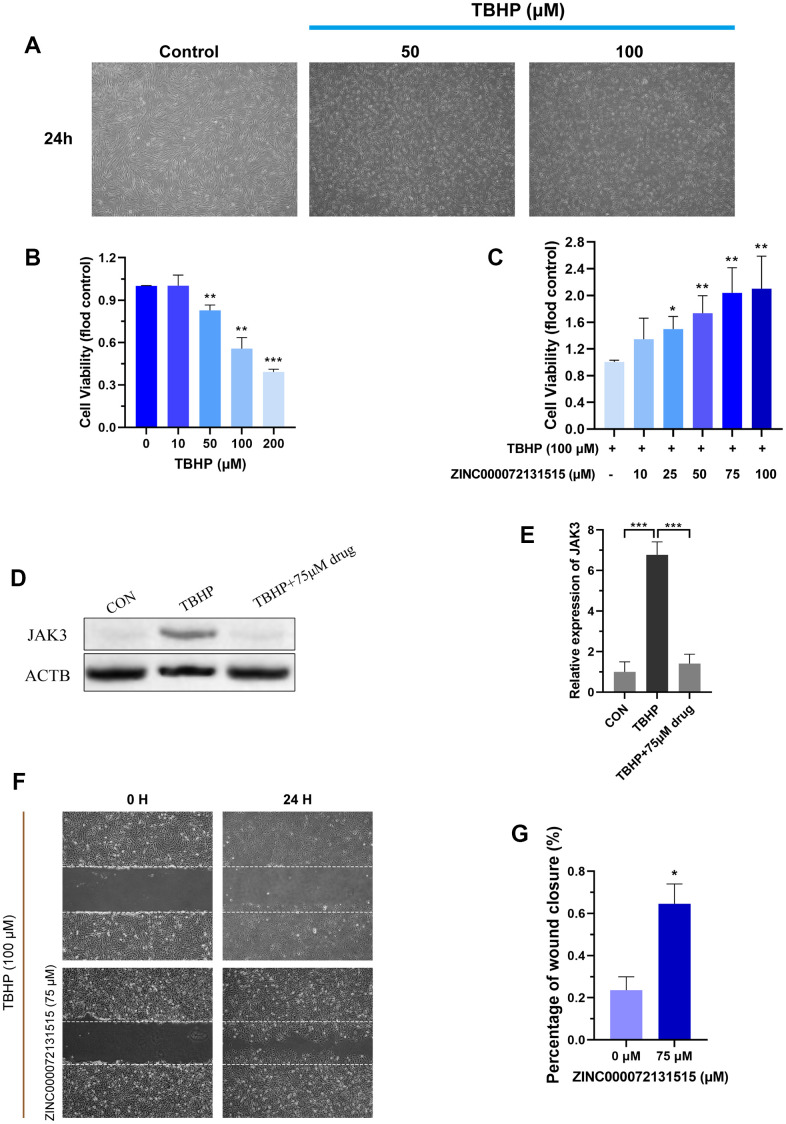
(**A**) NP cells were treated with different concentrations (50, 100 μM) of TBHP after 24 h and imaged by phase-contrast microscopy. (**B**) Cellular viability of the NP cells with different concentration of TBHP for 24 h. (**C**) Cellular viability of the TBHP-induced (100 μM) NP cells with different concentration of ZINC000072131515. (**D**, **E**) The protein expression of JAK3 in NP cells treated with different concentrations of ZINC000072131515. (**F**) NP cells migration was determined by scratch experiments, the results were recorded at 0, 24 h. (**G**) Percentage of wound closure of NP cells at 24 h. *P < 0.05; **P < 0.01; ***P < 0.0001; ns, non-significance.

After 100μM TBHP treatment for 24h, the volumes of NP cells shrank, with floated cells and dead cells increasing (Figure 10A, 10B). Besides, the cellular viability of NP cells was restored with different concentration of ZINC000072131515 treatment in degenerative NP cells (0, 10, 25, 50, 75, 100μM), which displayed evident protective roles and reversed the TBHP-induced NP cell apoptosis (Figure 10C). Besides, the expression of JAKs family was further detected by western blot analysis. The expression of JAK3 protein was inhibited by ZINC000072131515 with concentrations increasing (Figure 10D, 10E), illustrating that ZINC000072131515 was a potential JAK3 inhibitor, which may provide more resource reserves for the current pharmaceutical market.

### ZINC000072131515 restored migration capacity of degenerative NP cells

After 100μM TBHP treatment for 24h for NP degeneration model establishment, we observed that TBHP significantly inhibited the migration of NP cells, while ZINC000072131515 could reverse the side effects and restored the migration capacity (Figure 10F, 10G).

## DISCUSSION

In this study, we used integrated analysis of bulk RNA-seq, scRNA-seq, as well as WGCNA analysis to comprehensively discover the initiation, development as well as the treatment approach of IDD. Currently, most studies selected the top 5000 genes based on the median absolute deviation for WGCNA workflow, due to the hashrate of personal computer. This study possessed high performance server, which could include all genes for analysis, so as to establish the comprehensive WGCNA network. Although 4 hub genes were discovered, the significance was more focused on the prediction, that meant these 4 genes were more suitable for predicting the occurrence of IDD. While through the KEGG analysis based on all the identified DEGs, we observed that JAK-STAT signaling pathway was significantly activated, as shown in Figure 2C. The generated signaling pathways obtained by including all DEGs were often more convincing, since the results were integrated by diversity genes. Besides, the JAK-STAT signaling pathway was reported to be closely connected with the IDD [10, 11], which was consistent with our findings in this study. Consequently, based on the aberrantly activated signaling pathway JAK-STAT, this study further performed high-throughput virtual screening technique to discover the potential nutrients that could ameliorate IDD.

JAKs families are tightly connected to the development in a variety of tumors and non-tumor diseases, among them, JAK3 is an essential regulatory gene, and its mediated signaling pathway keep highly active in these diseases. Recent researches have gradually focused on the relationships between JAKs and bone-related diseases like OA, OP and IDD [10, 11, 18, 34]. And natural materials discovery based on JAKs family might also advance the development of potential compounds in the treatment of bone-related diseases. Due to the selectivity of the binding between JAK3 and interleukin receptors, JAK3 has the most unique functions in cytokines transduction among JAKs family [48]. Besides, the roles of JAK3-STAT3 axis in bone homeostasis also prompted more researches focusing natural materials discovery based on genetic therapy [21]. Therefore, taking JAK3 as a regulatory gene and discovering strategies to effectively suppress its functions have become a hotspot in recent years.

The existed targeted molecules with immunosuppressants are widely distributed and have poor selectivity, which could lead into multi-directional adverse effects. Current discovered JAK3 inhibitors have low selectivity, which could cause complications like thrombocytopenia, leukopenia and anemia [7]. Peficitinib has showed promising efficacy in ameliorating symptoms of skeletal disorders, with acceptable tolerance, which is a selective inhibitor of JAK3; however, the side effects are also obvious in infections and infestations [49, 50]. FM-381, as a newly reported covalent-reversible JAK3 inhibitor recently, the interaction modes between molecules have been well-studied [21]; while it still has some limitations that its bioavailability and indicators like pharmacological properties have not been conducted well. Consequently, more ideal candidate targeted compounds need to be conducted to fulfil the gap of JAK3 inhibitors, which also requires structure optimization and modification on pharmacophore to enhance the selectivity of the inhibitor and reduce toxicity. Thus, this study selected FM-381 as the reference compound, to discover more potential compounds referred to the binding pattern of FM-381 with JAK3.

In this study, huge number of ligands were prepared and generated an amount of small molecules in ZINC15 database through high throughput virtual screening. Higher Libdock scores indicated more stable posture. According to the Libdock scores, the top 50 compounds were screened for further analysis. ADME predictions indicated that ZINC000072131515, ZINC000014952116 and ZINC00000393864 predicted to have no hepatotoxicity, indicating that they were relatively safe drugs and would cause little damage to the liver when long time usage. Then, toxicity predictions suggested ZINC000072131515, ZINC000014952116 and ZINC000003938642 obtained less Ames mutagenicity, rodent carcinogenicity as well as DTP potential, suggesting that they were less harmful. Molecular dynamics simulation results further illustrated that the curves of these three complexes including energy values and RMSD values of these complexes attained equilibrium with the time. Therefore, they were considered to have existed stably under *in-vivo* situation. Briefly, the advantages of these three compounds were determined as potential lead compounds.

The initial discovery of potential lead compounds is a key step in the following drug design and development. To investigate the effects of these compounds in IDD, we bought ZINC000072131515 for further *in-vitro* experiments. Our results suggested that ZINC000072131515 was able to inhibit TBHP-induced apoptosis of NP cells in a dose-dependent manner, indicating the promising approaches based on the targeted macromolecules.

Considering the characteristics of ZINC000072131515, ZINC000014952116 and ZINC000003938642, these compounds possessed lots of editable cites, the pharmacophore modification site analysis suggested that based on these skeletons, the pharmacological properties could be improved to reduce its adverse effects, toxicity and improve its stability, by embellishing its specific functional groups or atoms, to continuously improve the pharmacological effects of potential compounds. In this regard, these three candidate compounds also provided novel skeletons for researchers to focus on and for more improvement analysis in the future.

Briefly, this study mainly applied advanced structural biological methods to discover ideal lead compounds, and also calculated the possible modification cites of pharmacophores on these candidate compounds. Among them, ZINC000072131515 served as a potential JAK3 inhibitor, exhibited promising effects to ameliorate the progression of IDD, which may provide more resource reserves for the current pharmaceutical market. Currently, only phenotypic validations of ZINC000072131515 were performed in this study, more *in-vivo* or *in-vitro* analysis should be conducted in the future, to provide the latest therapeutic strategies and molecular mechanisms for alleviating IDD.

## Supplementary Material

Supplementary Figures
