# Supplementary material for: Conjoint research of WGCNA, single-cell transcriptome and structural biology reveals the potential targets of IDD development and treatment and JAK3 involvement
Source: Aging (Albany NY). 2023 Dec 12;15(24):14764–90. doi: 10.18632/aging.205289 (PMC10781489; doi:10.18632/aging.205289)
Supplement: Supplementary Figures [file aging-15-205289-s001.pdf]

SUPPLEMENTARY FIGURES

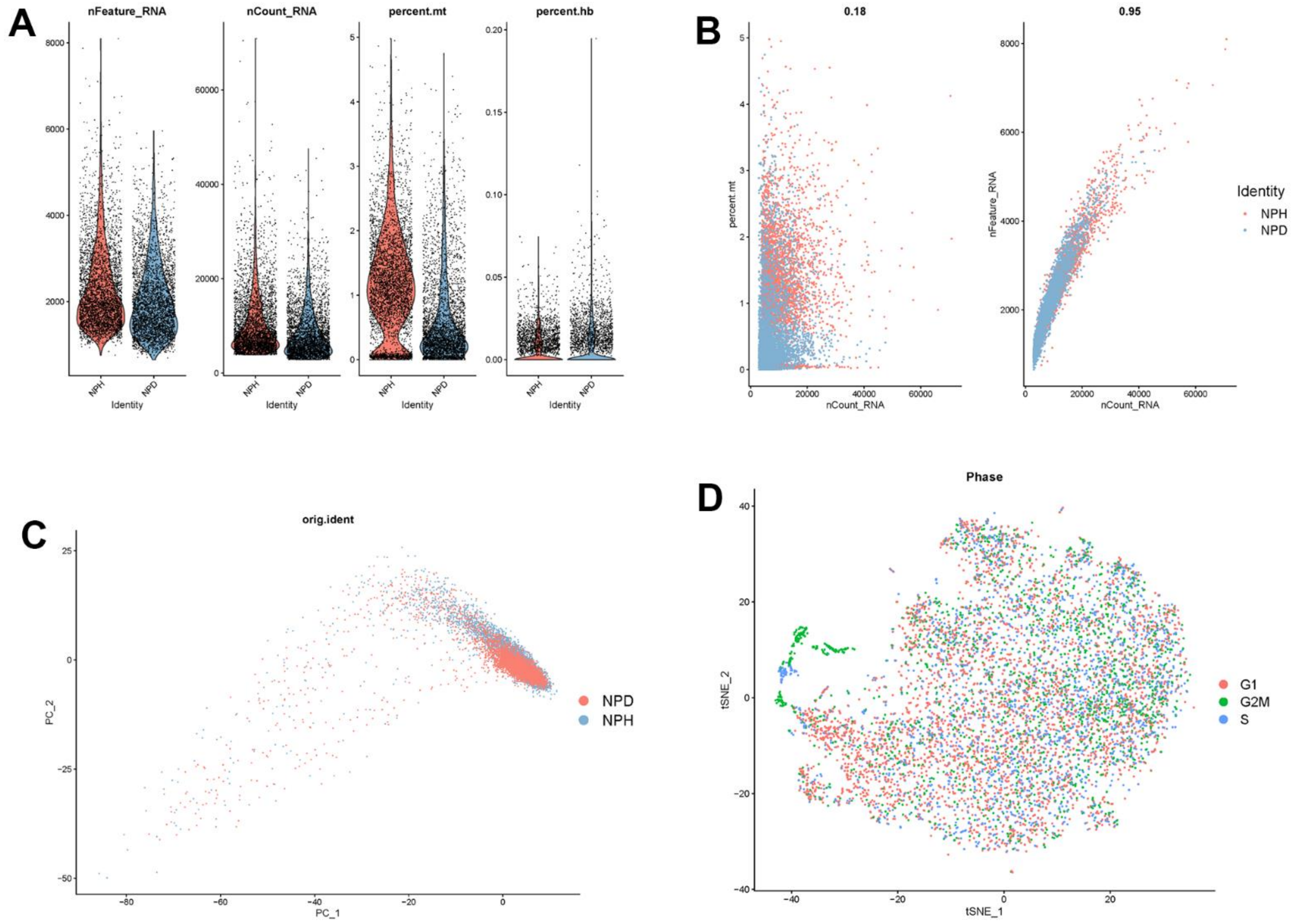

Supplementary Figure 1. Quality check process of the scRNA-seq analysis. (**A**) Violin plot showing the existed cells after quality control. (**B**) The left panel indicated the mitochondria UMI rate of each cell, the right panel suggested the correlations between the genes and sequencing depth. (**C**) Scatter plot of the cells distribution after batch effects elimination. (**D**) Scatter plot of the cycle's distribution after cell cycle-related genes integration.

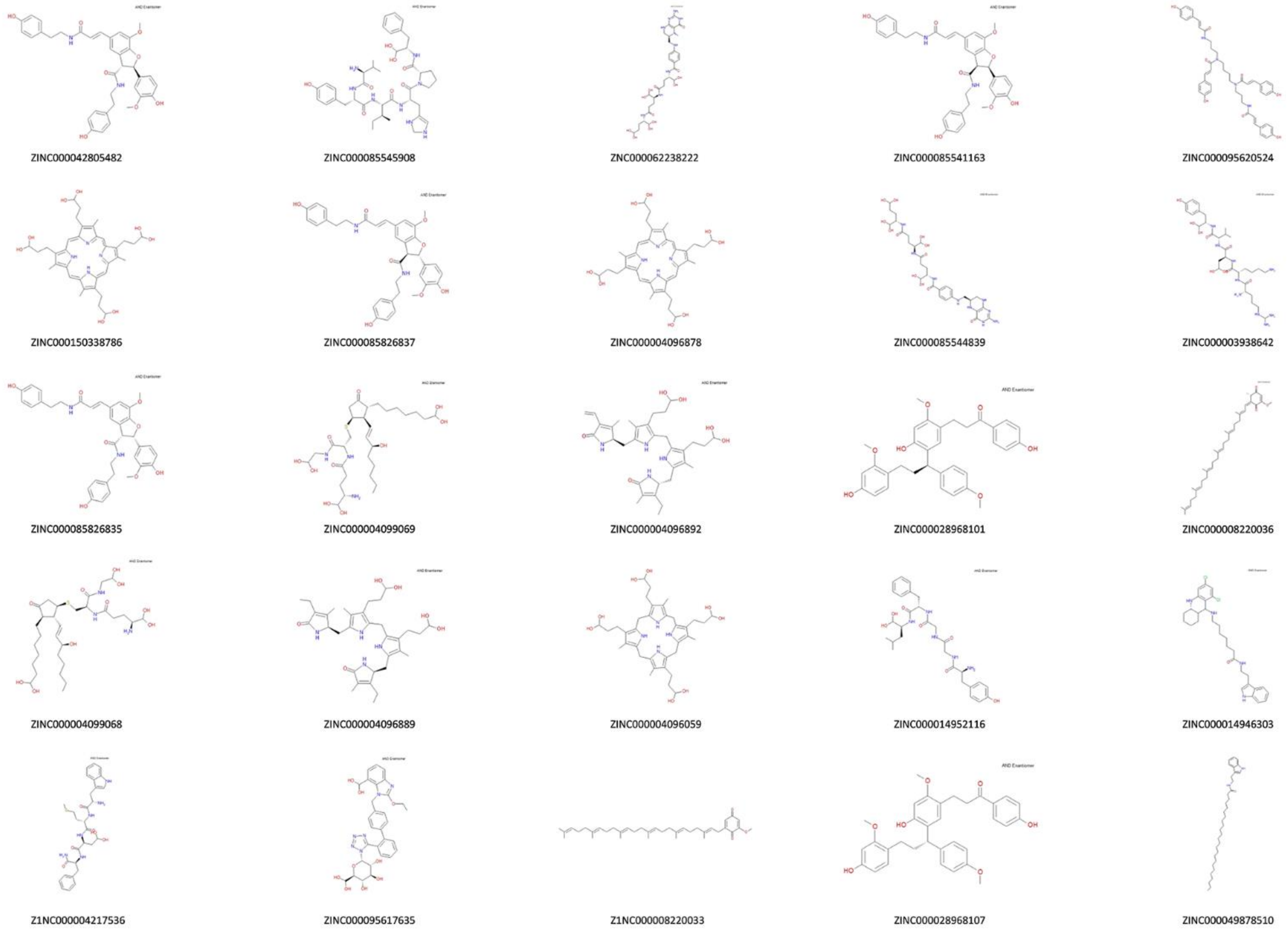

Supplementary Figure 2. The chemical structures of the top 20 compounds by Libdock module.
